# The cascading effects of human food on hibernation and cellular aging in free-ranging black bears

**DOI:** 10.1038/s41598-019-38937-5

**Published:** 2019-02-21

**Authors:** Rebecca Kirby, Heather E. Johnson, Mathew W. Alldredge, Jonathan N. Pauli

**Affiliations:** 1 Department of Forest and Wildlife Ecology, University of Wisconsin – Madison, 1630 Linden Dr., Madison, WI 53706 USA; 2 Mammals Research Section, Colorado Parks and Wildlife, 415 Turner Dr., Durango, CO 81303 USA; 3 Present Address: USGS Alaska Science Center, 4210 University Dr., Anchorage, AK 99508 USA; 4 Mammals Research Section, Colorado Parks and Wildlife, 317 W. Prospect Rd., Fort Collins, CO 80526 USA

## Abstract

Human foods have become a pervasive subsidy in many landscapes, and can dramatically alter wildlife behavior, physiology, and demography. While such subsidies can enhance wildlife condition, they can also result in unintended negative consequences on individuals and populations. Seasonal hibernators possess a remarkable suite of adaptations that increase survival and longevity in the face of resource and energetic limitations. Recent work has suggested hibernation may also slow the process of senescence, or cellular aging. We investigated how use of human foods influences hibernation, and subsequently cellular aging, in a large-bodied hibernator, black bears (*Ursus americanus*). We quantified relative telomere length, a molecular marker for cellular age, and compared lengths in adult female bears longitudinally sampled over multiple seasons. We found that bears that foraged more on human foods hibernated for shorter periods of time. Furthermore, bears that hibernated for shorter periods of time experienced accelerated telomere attrition. Together these results suggest that although hibernation may ameliorate cellular aging, foraging on human food subsidies could counteract this process by shortening hibernation. Our findings highlight how human food subsidies can indirectly influence changes in aging at the molecular level.

## Introduction

Human food subsidies, like garbage, crops, and livestock, are a ubiquitous consequence of human development^1–3^. While such food subsidies can enhance nutritional condition and physiological performance of wildlife^4^, more human food may not always be better. Easily accessible human foods may lack species-specific nutritional requirements^5,6^, contain lethal toxicological compounds^7^, or enhance the spread of disease^8^. Consumption of human foods can also alter animal behavior^9^, increasing the risk of injury or mortality in human-dominated landscapes^10,11^. In general though, the consequences of human food subsidies on the individual fitness and longevity of free-ranging animals remain largely unknown.

Torpor, a state of lowered metabolic demand, has evolved as an adaptive response to food limitations and harsh environmental conditions. Although the degree and type of torpor range widely across animal groups, one of the deepest and most extended forms is seasonal hibernation^12^, which is observed in eight groups of mammals. By lowering body temperatures and reducing metabolic rates, hibernators accrue significant energetic savings and avoid predation, which increases overwinter and annual survival^13^, with direct implications for longevity^14^. In particular, small-bodied mammals that can enter hibernation possess lifespans longer than expected from their body size or metabolic rate^15^. This increased longevity appears to have coevolved with aspects of a relatively slow life history strategy, including delayed onset of senescence^13,16^. Hibernation, then, not only conserves energy, but may also be adaptive in slowing cellular aging^14^. Increasingly, researchers are utilizing telomeres – repetitive DNA sequences on the ends of eukaryotic chromosomes^17,18^ that are lost during cellular replication and from oxidative damage^19^ – as markers to quantify cellular aging, or aging distinct from chronology^20–22^. Recent studies have found that more time spent in torpor can decelerate telomere attrition, or reduce cellular aging, among small hibernators^23–25^. Although the exact mechanism of hibernation that slows cellular aging in small-bodied mammals is unknown, it appears to be associated either with a reduction in cell turnover rates^26^ or a reduction in oxidative stress^24^.

Changes to hibernation strategies and characteristics, then, are likely to have important implications for individual fitness. For example, warmer weather during the winter and spring due to climate change^27^ has altered the timing of emergence, leading to phenological mismatches with food sources^28^ and reducing individual fitness^29^. Expanding human development and increased wildlife access to supplemental food has been linked to delayed or shortened hibernation^11,30,31^, and even the loss of hibernation for a winter altogether^32^. Shortened hibernation periods are likely to lead to similar mismatches with local food sources and increased interactions and conflicts with humans^11,31^. It is unknown what these consequences will have on individual physiology or fitness traits, but given that hibernation is modulated primarily by local food conditions^11,12,30,33^, natural food availability and human subsidies could indirectly govern senescence by altering rates of cellular aging.

In this study, we investigated the relationship between food subsidies, hibernation, and cellular aging in the American black bear (*Ursus americanus*). As large-bodied hibernators, bears are sufficiently long-lived to exhibit senescence^34,35^, but unlike small hibernators, they remain near-euthermic during hibernation in spite of their reduced metabolic rate^36^ and increased oxidative stress^37^. Preliminary research suggests that cellular aging in black bears is driven principally by environmental conditions—such as natural food availability—found at different latitudes^38^. Bears generally hibernate for 4–6 months/year, and denning chronology is driven in part by forage availability – individuals with access to more food tend to enter hibernation later and den for shorter periods^11,30,39^. Furthermore, black bears often supplement their diet with human food subsidies, especially in years of natural food shortages^40,41^. Bears that use areas of human development show decreased hibernation periods^11,30,31^. This altered denning chronology is assumed to result from increased consumption of food subsidies, although this link has not been directly explored. To assess the effects of food subsidies on hibernation and cellular aging, we tracked and sampled a subset of female black bears through several summer and winter seasons as part of a larger study in Durango, Colorado, USA^11,40^. We analyzed bear stable isotopic signatures (δ^13^C) as a measure of consumption of human foods^41,42^, and determined the influence of use of human food on hibernation lengths across individuals. We then assessed the relationship between hibernation length and rates of telomere length to test the role of hibernation in cellular aging. Finally, we examined whether the specific role of oxidative stress associated with hibernation is a potential mechanism mediating telomere length change in bears.

## Results

Female black bears (*n* = 30) averaged 8 years old at first sampling (range: 2 to 24) and hibernation lengths over the study averaged 170 days (range: 134 to 223). Summer sampled bears averaged −20.63 δ^13^C (range: −22.36 to −18.80). Bear serum exhibited average oxidative damage of 10.8 mg H_2_O_2_ dl^−1^ (range: 4.5 to 18.8) and average antioxidant capacity of 516 μmol HClO ml^−1^ neutralized (range: 349 to 769). Age was positively correlated with hibernation length (*r* = 0.73, *P < *0.001); however, given the importance of age in determining bear physiology and behavior^11^, and that the variance inflation factor was only 1.47, we retained age as a covariate in subsequent tests.

**Figure 1:**
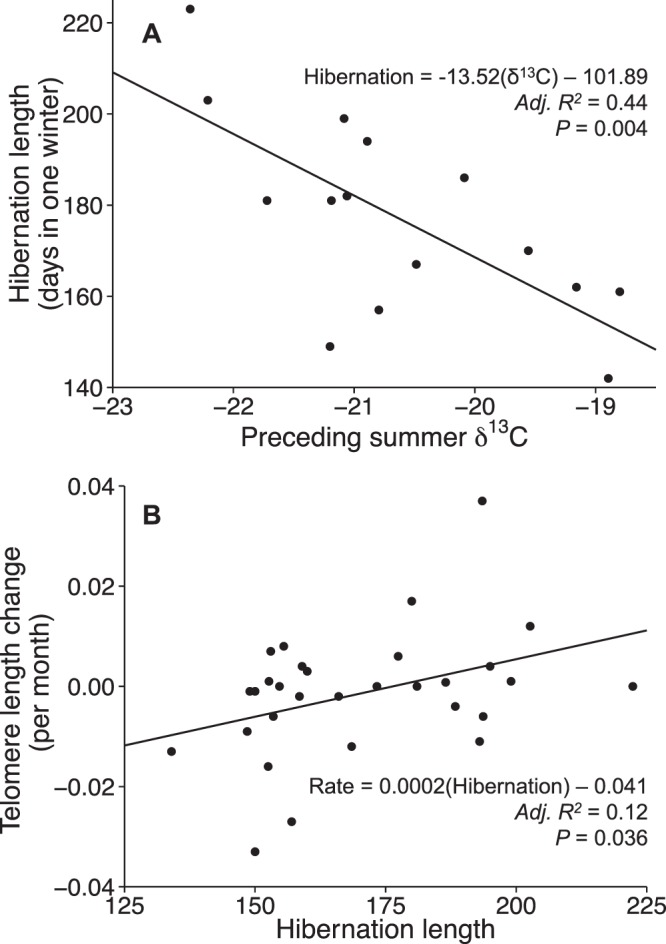
(**A**) Hibernation length for each bear (over one winter) regressed on the δ^13^C signature of bear hair sampled in the preceding summer (*n* = 15), showing a relationship between increased enrichment in δ^13^C and shorter hibernation lengths. (**B**) Average monthly telomere length (RTL) change regressed against hibernation lengths (days) for each bear (*n* = 30), exhibiting a relationship between longer hibernation length and slower rate of telomere shortening, and even telomere lengthening.

Bears enriched in δ^13^C during the summer (i.e., those that consumed more human foods), as well as younger bears, hibernated for shorter periods the subsequent winter (Table 1a, Fig. 1A). Telomere lengths on average decreased at a rate of 0.001 RTL/month (*σ* = 0.01) throughout the study period, but this pattern was inconsistent, as almost half the bears showed increased telomere lengths. We found that the mean monthly rate of telomere change was related to hibernation length; bears that hibernated longer on average experienced a slower rate of telomere attrition or even telomere lengthening during the study (Table 1b, Fig. 1B). There was limited support that telomere length change was related to oxidative stress (antioxidant capacity/oxidative damage; Table 1b; model coefficients are reported in Supplementary Table 1).

**Table 1:** Models ranked by AIC_c_ to predict: (a) hibernation length over one winter, with age and δ^13^C signature of bear hair sampled in the preceding summer as covariates (*n* = 15); (b) average monthly telomere length change, with age, oxidative stress, and hibernation length over the study period as covariates (*n* = 30).

	AIC_c_	ΔAIC_c_	weight	Adj. R^2^
**(** ***a*** **)** ***Hibernation length***				
δ^13^C	132.48	0.00	0.60	0.44
δ^13^C + Age	134.26	1.78	0.25	0.47
Age	135.57	3.09	0.13	0.32
Intercept only	139.19	6.71	0.02	
**(** ***b*** **)** ***Telomere length change*** **(** ***per month*** **)**				
Hibernation	−176.08	0.00	0.39	0.12
Age	−174.38	1.70	0.17	0.07
Intercept only	−173.76	2.32	0.12	—
Hibernation + Oxidative stress	−173.49	2.60	0.11	0.09
Hibernation + Age	−173.49	2.60	0.11	0.09
Age + Oxidative stress	−172.00	4.09	0.05	0.04
Oxidative stress	−171.40	4.68	0.04	0.00
Hibernation + Age + Oxidative stress	−170.70	5.38	0.03	0.06

Oxidative damage (ROM) was related to sampling season, breeding status, and age (Table 2a). Bears exhibited increased oxidative damage during hibernation compared to the summer, and bears that had newborn cubs exhibited reduced oxidative damage compared to those that were barren or had yearlings. We found minimal differences in antioxidant capacity among bears based on our covariates (model coefficients are reported in Supplementary Table 2).

**Table 2:** Models ranked by AIC_c_ to predict measures of: (a) oxidative damage (ROM) and (b) antioxidant capacity in black bear serum (unique bears = 28 and samples = 84, with repeated bear samples accounted for with a random effect. Fixed effects included age, season (active/summer or hibernation/winter), and reproductive status at summer sampling (barren or with cubs, as yearlings had already dispersed).

	AIC_c_	ΔAIC_c_	weight	marginal *R*^2^	conditional *R*^2^
**(** ***a*** **)** ***Oxidative damage***					
Age + Season + Breeding status	506.36	0.00	1.00	0.26	0.42
Age + Breeding status	519.39	13.03	0.00	0.15	0.41
Age + Season	519.54	13.18	0.00	0.13	0.34
Age	531.17	24.81	0.00	0.04	0.27
Season + Breeding status	554.39	48.03	0.00	0.21	0.33
Breeding status	567.09	60.74	0.00	0.10	0.31
Season	567.21	60.85	0.00	0.10	0.27
Intercept only	578.20	71.84	0.00	—	—
**(** ***b*** **)** ***Antioxidant capacity***					
Age + Season + Breeding status	1011.64	0.00	0.96	0.01	0.53
Age + Breeding status	1017.88	6.24	0.04	0.01	0.54
Age + Season	1025.29	13.65	0.00	0.00	0.53
Age	1031.29	19.66	0.00	0.00	0.53
Season + Breeding status	1115.15	103.52	0.00	0.01	0.54
Breeding status	1121.19	109.55	0.00	0.01	0.55
Season	1128.93	117.29	0.00	0.00	0.54
Intercept only	1137.79	126.16	0.00	—	—

## Discussion

Highly accessible and predictable food subsides can alter animal behavior^9,43^, change population dynamics^44^, and restructure community assemblages and species interactions^45,46^. Our study demonstrates that such food subsidies are also associated with cellular aging indirectly via altering hibernation length. Black bears with a greater reliance on human food subsidies were associated with having shorter hibernation lengths, and these shortened hibernation periods were associated with greater telomeric attrition. Consequently, bears that use more food subsidies hibernate less and thereby appear to experience greater cellular aging.

Hibernation chronology is driven by individual energy balance^47^, which is strongly linked to local weather conditions and food availability^11,30^. Recent work has shown that bears with access to more food, and bears exhibiting increased use of human development, den later and for a shorter period^11,31^. Our results demonstrate that greater consumption of human foods is associated with shorter hibernation in black bears. Increased consumption of human foods by bears has been associated with increased body weights and fecundity, but also reduced survival (due to vehicle collisions, lethal management, etc.)^10,48^. As a result, it has been suggested that urban areas may serve as an ecological trap^10,41^. This risk may be compounded by increased bear-human interactions resulting from shortened denning^31^, as well as have further consequences on fitness, through altered hibernation and accelerated telomere loss.

Bears display a remarkable suite of adaptations allowing them to remain immobile during hibernation, yet avoid negative side effects such as bone loss^49^ and muscle atrophy^50^. An additional advantage of hibernation appears to be slowed cellular aging; we found that bears with longer average hibernation lengths showed reduced rates of telomere shortening over the study period. Our finding corroborates recent work in small hibernators that effectively demonstrated that longer and deeper bouts of torpor slowed cellular aging^23–25^. Because telomere dynamics reflect accumulated life stress^20^ and can predict survival and longevity^21^, altering those dynamics through shortened denning periods may have negative long-term consequences.

Some animals display adaptations to counteract telomeric shortening, such as unusually high levels of the enzyme telomerase, which lengthens telomeres^51,52^. Although oxidative damage is typically an accelerant of telomere attrition^19,53,54^, animals that increase their antioxidant capacity might be able to mitigate such effects^55^. However, we found that although bears exhibited increased oxidative damage during hibernation compared to the active season^37^, we did not detect a concurrent increased antioxidant capacity. According to these stress measures, it appears that hibernation ameliorates cellular aging in spite of increased oxidative damage, perhaps due to reduced metabolic rate or enhanced somatic maintenance. This lack of a relationship between oxidative stress and telomere attrition could, however, also be influenced by our sampling - telomeres were not measured immediately before and after hibernation, and therefore may be more representative of stress experienced throughout the study period, not only during hibernation.

In addition to seasonal differences, oxidative damage differed among breeding status; females with cubs showed less damage, corroborating a recent study in polar bears^56^. Reproduction, and lactation in particular, is energetically expensive^57,58^, and resulting oxidative stress is typically regarded as a cost of reproduction^59^. The relationship between reduced oxidative damage and reproduction in bears remains unclear; however, researchers have speculated it could result from physiological changes during lactation that allow the off-loading of contaminants that otherwise induce oxidative stress^56^.

Our study of a free-ranging large hibernator suggests that increased reliance on human food subsidies reduces hibernation lengths. Our study also supports previous work on small hibernators that a benefit of hibernation is decelerated telomere attrition^23^. Thus, bears consuming more human foods may lose some of the long-term fitness advantages associated with hibernating, in particular rates of cellular aging. Therefore, the continued growth in food subsidies to wildlife are likely to cascade into altered behavior, ultimately with potential molecular consequences for rates of cellular aging.

## Methods

### Sample collection

Black bears were captured near Durango, Colorado, from summer 2011 through winter 2015. All captures and animal handling were performed in accordance with relevant guidelines and regulations and approved by Colorado Parks and Wildlife [CPW], Fort Collins, CO (Animal Care and Use Protocol #01-2011)^11^. Adult females were fitted with GPS collars (Vectronics Globalstar) and subsequently relocated at their winter dens. Thirty bears were included in this study that were sampled a minimum of twice during the study period, twenty-six were sampled ≥3 times. Sampling occurred during initial capture in summer (mainly June – August) and then again during winter den visits (mainly early February – mid-March) in subsequent years; 18 of the bears were sampled in both the summer and winter within the same year.

During captures, bears were immobilized^11^, and guard hair and blood samples were collected for molecular analyses. At first capture, a premolar was removed to determine chronological age by counting cementum annuli (Matson’s Lab, Milltown, MT)^60^. Breeding status was also identified by the presence/absence of cubs (or lactation during summer captures when cubs were not always visible) or yearlings, and adult females categorized as “with yearlings”, “with cubs”, or “barren”. Black bear cubs are born during hibernation, and nurse part of that first year, typically staying with their mother through the next winter season; at the start of the second summer, yearlings will disperse.

We used collar activity sensor data to determine den entry and exit dates for each bear on an annual basis^11^. In 11 observations (out of 58 total), activity data were not available to estimate denning dates. In those cases, we used hourly GPS locations to define den entry as the first day of a 6-day period when a bear was exclusively located within 135 m of her den, and den emergence as the first day of a 6-day period when a bear remained 135 m away from her den^61^. Hibernation length was calculated as the number of days between den entrance and emergence.

### Laboratory analyses

Blood samples for DNA extraction were stored in EDTA tubes; those for oxidative stress analyses were kept in serum-separating tubes. All samples were stored at −20 °C until analyses. We extracted DNA with standard procedures (QIAGEN DNeasy Blood and Tissue Extraction Kit; QIAGEN Inc., Valencia, CA). We quantified relative telomere lengths (RTL) using real-time quantitative polymerase chain reaction (qPCR)^62^. We previously optimized this method using the HNRPF gene^63^ and telomere primers telg and telc^38,64^ (Supplementary Material). We quantified relative telomere lengths from each sample. Because samples were collected once in the summer, and following mid-winters, we accounted for differences between sampling times of individuals by calculating an overall telomere length change for each bear between their first and last capture, averaged over months (*n* = 30).

Hair samples were prepared for stable isotope analyses as described in Pauli *et al*. 2009^65^. Results are provided as per mil (‰) ratios relative to international standard, with calibrated internal laboratory standards. Individual foraging was represented by δ^13^C of hair samples; specifically enrichment in δ^13^C signifies increased human food in bear diets^41,66^. Human foods are enriched in δ^13^C compared to temperate native vegetation because they are dominated by corn and cane sugar derivatives^67^. Hair samples represent the assimilated diet during hair growth from spring through fall^68^, though in black bears tend to be highly correlated with stable isotopes in bone collagen, representing overall lifetime diet^42^.

We measured oxidative damage in bear serum samples, using the d-ROM test (Diacron International, Italy). The d-ROM test measures oxidative damage via the concentration of hydroperoxide, a reactive oxygen metabolite (ROM) that results from an attack of reactive oxygen species on organic substrates (e.g. nucleotides, proteins). The oxy-adsorbent test measures the total antioxidant capacity of the sample by measuring the ability of the serum to oppose the massive oxidative action of a hypochlorous acid (HClO) solution. Oxidative stress or status of an individual sample can be considered the ratio of antioxidant capacity to oxidative damage^55,69^. We prepared samples following the manufacturer’s protocol (Supplementary Material).

### Data analyses

We tested three main hypotheses: (1) bear consumption of human foods reduces hibernation length; (2) reduced hibernation accelerates telomere attrition (i.e., the cellular aging process); (3) increased oxidative stress is a mechanism mediating telomere attrition. To test whether foraging on human food subsidies influenced hibernation length, we used linear regression with hibernation length (days) as the response variable and δ^13^C of bear hair (sampled in the preceding summer) as an explanatory variable. We also included age as a covariate, to account for the fact that older bears hibernate longer^11^. We restricted our data to bears sampled in summer and then again in the following winter (*n* = 15). To test our second and third hypotheses, we explored the relationship between telomeres (rate of telomere change for each individual, standardized as change per month), hibernation length (days within one season for each individual, averaged over multiple seasons), and oxidative stress (ratio of antioxidant capacity to oxidative damage for each individual, averaged over the sampling period; *n* = 30). Finally, because repeated oxidative stress samples from an individual bear fluctuated throughout the study period, we also examined factors associated with individual measures of oxidative stress (oxidative damage and antioxidant capacity) during sampling, rather than averaged over the study. We examined separately how oxidative damage or antioxidant capacity varied with age, sampling season (summer or winter), and breeding status of bears with linear mixed models; repeated samples from the same bear were accounted for with a random effect (unique bears = 28, samples = 84). For all analyses, we compared linear regression models using Akaike’s Information Criteria corrected for small sample sizes (AIC_c_). The datasets are available from the corresponding author on reasonable request.

## Supplementary information

Supplementary Material
