# Supplementary material for: The cascading effects of human food on hibernation and cellular aging in free-ranging black bears
Source: Sci Rep. 2019 Feb 21;9:2197. doi: 10.1038/s41598-019-38937-5 (PMC6385323; doi:10.1038/s41598-019-38937-5)
Supplement: Supplementary file 1 — Supplementary Material [file 41598_2019_38937_MOESM1_ESM.pdf]

Supplementary Material

The cascading effects of human food on hibernation and cellular aging in free-ranging black bears

Rebecca Kirby[a*], Heather E. Johnson[b,c], Mathew W. Alldredge[d], Jonathan N. Pauli[a]

[a]Department of Forest and Wildlife Ecology, University of Wisconsin – Madison, 1630 Linden Dr., Madison, WI 53706, USA

[b]Mammals Research Section, Colorado Parks and Wildlife, 415 Turner Dr., Durango, CO 81303, USA

[c]Present address: USGS Alaska Science Center, 4210 University Drive, Anchorage, AK, 99508, USA

[d]Mammals Research Section, Colorado Parks and Wildlife, 317 W. Prospect Rd., Fort Collins, CO 80526, USA

*Corresponding author: Email: kirbyr@gmail.com, Tel: +1 608 215 2203, Fax: +1 608 262 9922

Supplementary Methods

Quantification of telomere lengths

We quantified relative telomere lengths (RTL) using real-time quantitative polymerase chain reaction (qPCR) [1]. This method determines relative telomere lengths by comparing the ratios of telomere repeat copy number (T) to a single copy/non-variable copy gene (S) in a DNA sample. Contrasting the T/S ratio allows the comparison of the relative differences in telomere length

between individuals (relative telomere length, RTL). Though any reliably amplified non-variable copy gene can be employed for standardization [2], we previously optimized this method using HNRPF gene [3] and telomere primers telg and telc [4,5].

DNA concentration was determined with Qubit 2.0 Fluorometer (Life Technologies) and DNA quality assessed using gel-electrophoresis. Telomere and single-copy gene PCR were conducted on separate 96-well plates, with identical preparation except for primers (see Kirby, Alldredge & Pauli 2017 for details). Each sample was analyzed in triplicate within a plate and the average used in subsequent analyses (coefficient of variations for T: within-plate = 14%, between-plate = 17%; S: within-plate = 8%, between-plate = 9%). Standard curves were generated from a mixture of 6 randomly chosen bear samples run in triplicate on each plate and diluted to 0.5, 1, 2.5, 6, and 10 ng/µl. Real-time PCR was conducted using an Eppendorf Mastercycler ep realplex, followed by baseline correction in LinRegPCR, and sample quantification using the standard curve method [6].

We examined relative telomere lengths at first sampling for each bear and calculated telomere length change (averaged over months, to account for differences in sampling time) within each individual between sampling periods and throughout the entire study (n = 30).

Oxidative stress analyses

We measured oxidative stress in bear serum samples, using the d-ROM and the oxy-adsorbent tests (Diacron International, Italy). The d-ROM test measures oxidative damage via the concentration of hydroperoxide, a reactive oxygen metabolite (ROM) that results from an attack of reactive oxygen species on organic substrates (e.g. nucleotides, proteins). Following the manufacturer's protocol, 1.5 µl of bear serum was mixed with 300 µl of an acidic buffer

solution, 3 µl of a chromogenic mixture, and incubated for 90 minutes at 37 °C. In these acidic conditions, iron is released from proteins catalyzing hydroperoxide to generate alkoxyl and peroxyl radicals, which react with the chromogenic mixture to produce a color intensity that is proportional to its concentration and read at 505 nm with a spectrophotometer. The concentration of hydroperoxide (expressed as mg H_2O_2 dl^{-1}) was calculated by comparison with a calibrator solution with an oxidative activity of 0.08 mg dl^{-1} (equivalent to that of H_2O_2). The oxy-adsorbent test measures the total antioxidant capacity of the sample by measuring the ability of the serum to oppose the massive oxidative action of a hypochlorous acid (HClO) solution. Briefly, serum was first diluted 1:100 with distilled water, 2 µl of the diluted sample was mixed with 200 µl of the oxidant (HClO-based) solution, and incubated at 37 °C for 10 minutes. After incubation, 2 µl of the chromogenic solution was added and the resulting color read with a microplate spectrophotometer at 505 nm, with the color intensity inversely related to the antioxidant capacity, expressed as µmol HClO ml^{-1} neutralized. For each assay, all samples were analyzed in triplicate and the averages were compared to standard solutions. The inter-assay coefficients of variation were 0.10 and 0.06 for d-ROM and oxy-adsorbent tests, respectively.

Stable isotope analyses

Hair samples were rinsed three times with 2:1 chloroform:methanol solution, homogenized with surgical scissors, and dried to 72 hours at 56°C [7]. Samples were then weighed into tin capsules and analyzed at University of New Mexico's Center for Stable isotopes using a Costech 4010 and Carlo Erba 1110 Elemental Analyzer (Costech, Valencia, CA) attached to a Thermo Finnigan Delta Plus XP Continuous Flow Isotope Ratio Mass Spectrometer (Thermo Fisher

Scientific Inc., Waltham, MA). Results are provided as per mil (‰) ratios relative to the international standard of Vienna Peedee Belemnite, with calibrated internal laboratory standards.

References

1. Cawthon, R. M. Telomere measurement by quantitative PCR. *Nucleic Acids Res.* **30,** e47 (2002).
2. Olsen, M. T., Bérubé, M., Robbins, J. & Palsbøll, P. J. Empirical evaluation of humpback whale telomere length estimates; quality control and factors causing variability in the singleplex and multiplex qPCR methods. *BMC Genet.* **13,** 77 (2012).
3. Fedorov, V. B. *et al.* Elevated expression of protein biosynthesis genes in liver and muscle of hibernating black bears (*Ursus americanus*). *Physiol. Genomics* **37,** 108–18 (2009).
4. Cawthon, R. M. Telomere length measurement by a novel monochrome multiplex quantitative PCR method. *Nucleic Acids Res.* **37,** e21 (2009).
5. Kirby, R., Alldredge, M. W. & Pauli, J. N. Environmental, not individual, factors drive markers of biological aging in black bears. *Evol. Ecol.* **31,** 571–584 (2017).
6. Ruijter, J. M. *et al.* Amplification efficiency: linking baseline and bias in the analysis of quantitative PCR data. *Nucleic Acids Res.* **37,** e45 (2009).
7. Pauli, J. N., Ben-David, M., Buskirk, S. W., Depue, J. E. & Smith, W. P. An isotopic technique to mark mid-sized vertebrates non-invasively. *J. Zool.* **278,** 141–148 (2009).

Supplementary Table 1 Coefficients from the top model (<2 ΔAIC_c) for predictors of: **a)** hibernation length over one winter, with age and $\delta^{13}C$ signature of bear hair sampled in the preceding summer as covariates (n = 15); **b)** average monthly telomere length change, with age, oxidative stress, and hibernation length over the study period as covariates (n = 30).

***a)** Hibernation length*

	Variable	β	95% CI
Model 1	Intercept only	-101.89	(-259.08, 55.30)
	$\delta^{13}C$	-13.52	(-21.12, -5.91)
Model 2	Intercept only	-45.27	(-219.67, 129.13)
	Age	1.30	(-0.62, 3.22)
	$\delta^{13}C$	-10.11	(-19.07, -1.15)

***b)** Telomere length change (per month)*

	Variable	β	95% CI
Model 1	Intercept only	-0.04	(-0.08, 0.0008)
	Hibernation	0.0002	(0.000004, 0.0004)
Model 2	Intercept only	-0.007	(-0.01, 0.0008)
	Age	0.0007	(-0.00008, 0.001)

Supplementary Table 2 Coefficients from the top model (<2 ΔAIC_c) for predictors of oxidative damage and antioxidant capacity in black bear serum (unique bears = 28 and samples = 84, with repeated bear samples accounted for with a random effect. Fixed effects included age, season (active/summer or hibernation/winter), and reproductive status (at summer sampling, yearlings had already dispersed).

	Oxidative damage		Antioxidant capacity	
Variable	*β*	95% CI	*β*	95% CI
Intercept	9.63	(6.83, 12.43)	488.93	(403.75, 574.11)
Age	0.03	(-0.19, 0.25)	0.78	(-6.02, 7.58)
Season (winter)				
Summer	-4.96	(-7.49, -2.43)	-5.6	(-63.03, 51.83)
Reproductive status (with cubs)				
Barren	3.65	(1.08, 6.22)	27	(-31.56, 85.56)
With yearlings	5.01	(1.87, 8.15)	7.5	(-62.53, 77.53)

Supplementary Figure 1 Oxidative damage and antioxidant capacity of bear serum samples, compared between active (summer) and hibernating (winter) seasons, and among reproductive status (at summer sampling, yearlings had already dispersed). Bears show increased oxidative damage in the winter and decreased damaged with cubs. Antioxidant capacity did not differ among categories.

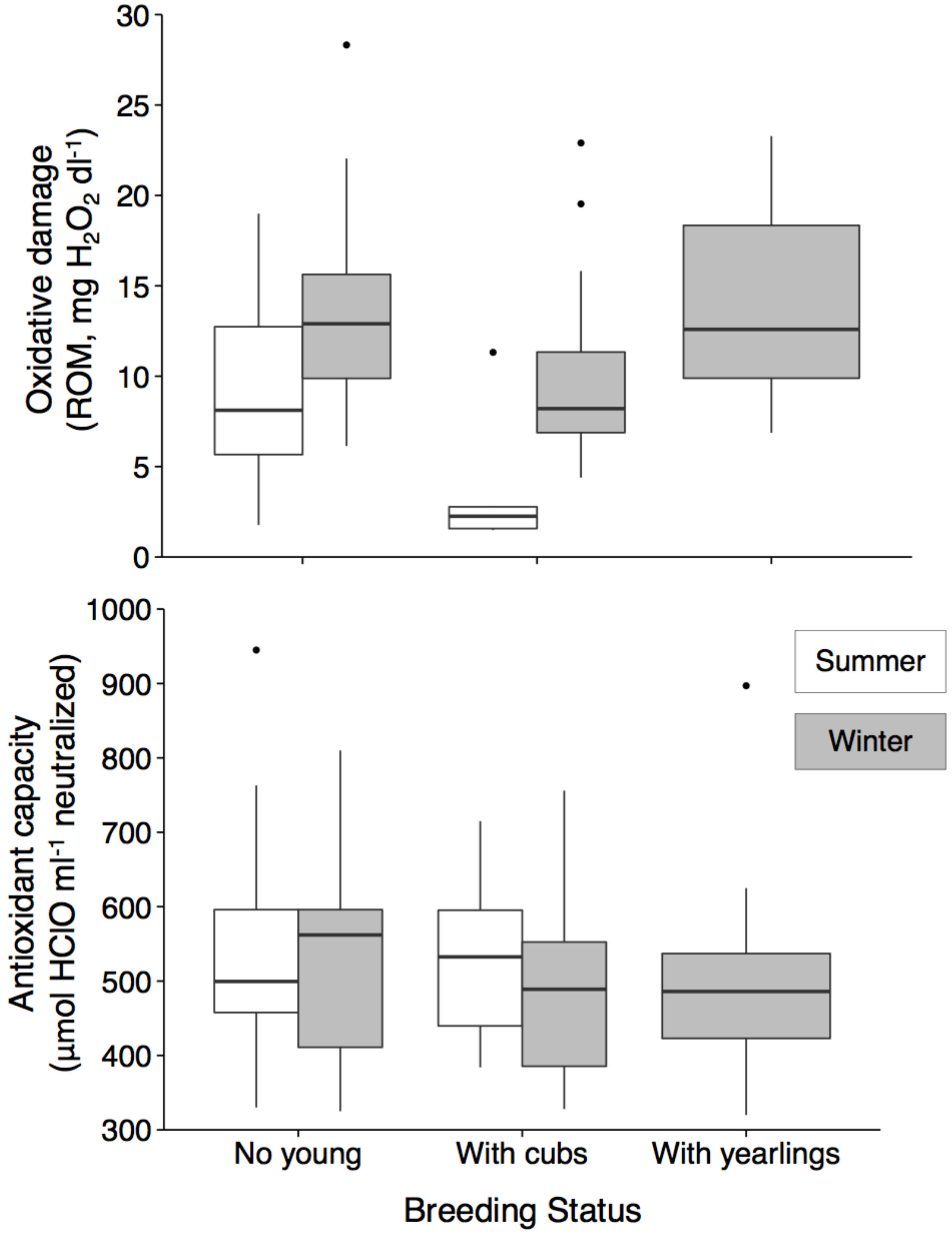

Oxidative damage (ROM, mg H_2O_2 dl^{-1})
30
25
20
15
10
5
0
Antioxidant capacity (μmol HClO ml^{-1} neutralized)
1000
900
800
700
600
500
400
300
Summer
Winter
No young
With cubs
With yearlings
Breeding Status
